# Ultrasound-Driven Defect Engineering in TiO_2–*x*_ Nanotubes—Toward Highly Efficient Platinum Single Atom-Enhanced Photocatalytic Water Splitting

**DOI:** 10.1021/acsami.3c04811

**Published:** 2023-07-25

**Authors:** Mahdi Shahrezaei, S. M. Hossein Hejazi, Hana Kmentova, Veronika Sedajova, Radek Zboril, Alberto Naldoni, Stepan Kment

**Affiliations:** † Czech Advanced Technology and Research Institute, Regional Centre of Advanced Technologies and Materials, Palacký University Olomouc, Slechtitelu 27, 77900 Olomouc, Czech Republic; ‡ Department of Physical Chemistry, Faculty of Science, Palacky University, 17. listopadu 1192/12, 77900 Olomouc, Czech Republic; § CEET, Nanotechnology Centre, VŠB−Technical University of Ostrava, 17. listopadu 2172/15, 70800 Ostrava-Poruba, Czech Republic; ∥ Department of Chemistry and NIS Centre, University of Turin, Turin 10125, Italy

**Keywords:** TiO_2_ nanotube arrays (TNTs), reduced TiO_2_, single-atom catalysts, hydrogen evolution (H_2_), photocatalysis

## Abstract

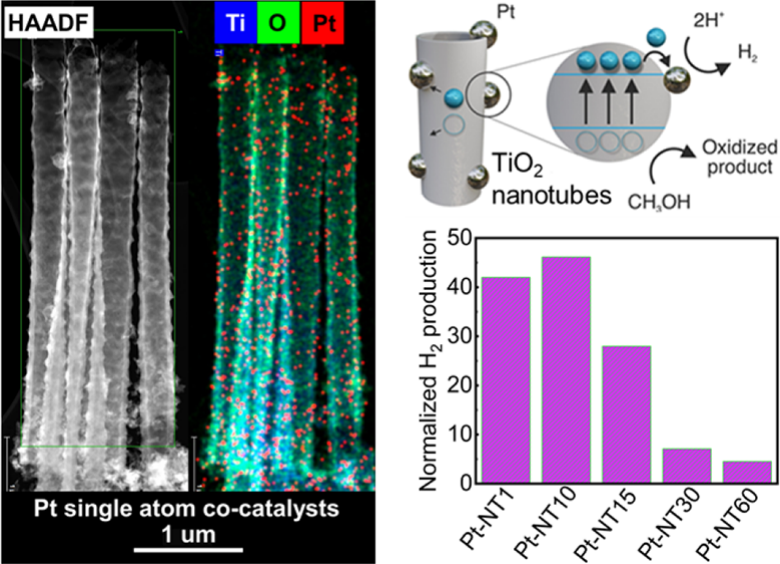

Single-atom catalysts (SACs) have demonstrated superior catalytic activity and selectivity compared to nanoparticle catalysts due to their high reactivity and atom efficiency. However, stabilizing SACs within hosting substrates and their controllable loading preventing single atom clustering remain the key challenges in this field. Moreover, the direct comparison of (co-) catalytic effect of single atoms vs nanoparticles is still highly challenging. Here, we present a novel ultrasound-driven strategy for stabilizing Pt single-atomic sites over highly ordered TiO_2_ nanotubes. This controllable low-temperature defect engineering enables entrapment of platinum single atoms and controlling their content through the reaction time of consequent chemical impregnation. The novel methodology enables achieving nearly 50 times higher normalized hydrogen evolution compared to pristine titania nanotubes. Moreover, the developed procedure allows the decoration of titania also with ultrasmall nanoparticles through a longer impregnation time of the substrate in a very dilute hexachloroplatinic acid solution. The comparison shows a 10 times higher normalized hydrogen production of platinum single atoms compared to nanoparticles. The mechanistic study shows that the novel approach creates homogeneously distributed defects, such as oxygen vacancies and Ti^3+^ species, which effectively trap and stabilize Pt^2+^ and Pt^4+^ single atoms. The optimized platinum single-atom photocatalyst shows excellent performance of photocatalytic water splitting and hydrogen evolution under one sun solar-simulated light, with TOF values being one order of magnitude higher compared to those of traditional thermal reduction-based methods. The single-atom engineering based on the creation of ultrasound-triggered chemical traps provides a pathway for controllable assembling stable and highly active single-atomic site catalysts on metal oxide support layers.

## 1 Introduction

Severe environmental pollution and energy crisis are among the main issues that have been given increasing attention by scientists during the past decade.^1−3^ The chemical conversion of molecular pollutants or small molecules such as water, carbon dioxide, and nitrogen into value-added chemicals is one of the most important strategies to tackle these urgent challenges.^4^ In this scenario, various homogeneous and heterogeneous catalysts have been designed to increase the rate of desirable reactions with higher product selectivity.^5^ Albeit homogeneous catalysts show higher activity and product selectivity, they suffer from difficult separation and recyclability.^6^ These disadvantages can be overcome using heterogeneous catalysts, which are indeed widely used in many industrial chemical reactions, but they have difficulty achieving more complex metal coordination. Single-atom catalysts (SACs) were proposed for the first time by Zhang in 2011^7^ and has recently emerged as a promising approach in catalysis, electrocatalysis, and photocatalysis combining the advantages of both homogeneous and heterogeneous catalysis, including the possibility to realize 100% of atomic utilization in catalytic reactions, easy separation from the reaction media, and unprecedented reaction yields/selectivities.^6,8^ Moreover, SACs allow the reduction of the content of precious metals such as Pt, Au, Rh, and Pd used in particular (photo-, electro-) catalytic reactions.^9,10^ The use of single-atom catalysts offers the access to ionic metallic species with enhanced reactivity compared to metallic species and enables achieving specific metal coordination and improved interaction with molecular species participating in the reaction.^11^ However, while single metal atoms exhibit increased reactivity, they also tend to aggregate, leading to the formation of either nanoclusters or nanoparticles (NPs). This aggregation causes again the loss of their exceptional catalytic activity.^11,12^ Furthermore, the strategies currently in use face challenges in controlling chemical traps and loading single atoms subsequently. For these reasons, one of the primary challenges in the field of SACs is the development of synthetic strategies that allow for stable and controllable anchoring of single atoms as well as homogeneous loading onto suitable support materials. This is crucial for achieving optimal catalytic performance and long-term stability.

Despite significant efforts in the field of photocatalysis, no photocatalyst has been reported yet that fulfills all the necessary criteria for efficient water splitting, including appropriate redox potential for water splitting, high photochemical stability, low electron–hole recombination rate, and visible light activation. In addition, there are further obstacles to achieving overall water splitting in pure water compared to sacrificial agent-assisted hydrogen evolution reactions. These obstacles include (i) uphill reaction (endothermic) from a thermodynamic standpoint; (ii) kinetically slow oxygen evolution reaction (OER) due to the unfavorable four-electron process required to produce an O_2_ molecule; (iii) evolution of oxygen bubbles on the surface of the photocatalyst after formation and growth; and (iv) backward reaction resulting in production of water, impeding the effective production of H_2_ and O_2_. To address these challenges, certain prerequisites can be considered for efficient H_2_ production in photocatalytic water splitting. These prerequisites include the use of co-catalysts, removal of O_2_, assistance from sacrificial agents, spatial separation of H_2_ and O_2_ in the photocatalytic setup, and the possibility of photocatalytic water splitting to produce both H_2_ and H_2_O_2_.^13^ Interestingly, it has been reported that noble metal co-catalysts can catalyze the backward reaction between H_2_ and O_2_. However, when noble metals are present in the form of single atoms, they hinder this recombination process, thus potentially improving the overall efficiency of water splitting.^14^

Various methods have been proposed to synthesize SACs, including bath deposition, vapor deposition, atomic layer deposition (ALD),^15−17^ co-precipitation,^18,19^ and wet-chemical synthesis such as impregnation and photo deposition.^20−22^ A promising and straightforward strategy for anchoring single atoms (SAs) onto suitable support materials involves nano- and atomic-scale defect engineering. This approach has shown significant potential for achieving stable and controllable anchoring of SAs, which is essential for optimizing their catalytic performance.^23−25^

Titanium dioxide (TiO_2_) is a widely studied photocatalytic material with great potential for producing solar fuels such as hydrogen and methanol through the use of sunlight to split water, carbon dioxide, and/or biomass like corn stover or sugarcane bagasse.^26−31^ This is due to numerous beneficial properties of TiO_2_, including its chemical and mechanical stability, resistance to photocorrosion, favorable band edge positions for photocatalytic hydrogen generation and charge transfer, rich surface chemistry that promotes the formation of various defects, and importantly, its low cost.^32−36^ Unfortunately, TiO_2_ suffers from a wide bandgap, high recombination rate of photogenerated electron–hole pairs, and low solar light absorption, which result in restricted practical photocatalytic applications.^37,38^

Surface decoration of TiO_2_ with foreign elements including non-metal (carbon, nitrogen, etc.) elements or noble metals like Pt, Pd, and Au is one of the most adopted strategies to improve the photocatalytic activity of TiO_2_ for photocatalytic degradation and hydrogen production.^23,38−40^ Lin et al. reported highly efficient Pd quantum dots with diameters of approximately 3.3 nm deposited on highly ordered TiO_2_ nanotubes for photoelectrocatalytic hydrogen production, which showed 1.6-fold higher activity than their conventional counterpart.^41^ Furthermore, these authors developed a hybrid structure of uniform nitrogen-doped carbon quantum dots anchored onto TiO_2_ and used it for degradation of organic contaminants and H_2_ production through water splitting.^42^ Among all the noble metals and from the electronic and catalytic points of view, Pt with the lowest overpotential and largest work function is the benchmark co-catalyst for H_2_ generation.^43^

However, as platinum is both scarce and expensive, significant efforts have been made to improve its efficiency in photocatalytic reactions. One effective method is reducing the particle size of the co-catalyst to the atomic scale, which not only makes it cost-effective but also maximizes its utilization efficiency.^11,44^ Several strategies have been reported for trapping Pt single atoms onto the surface of titanium dioxide to enhance photocatalytic processes. For instance, Hejazi et al.^23^ recently reported a method for depositing and stabilizing Pt single atom co-catalysts onto the surface of TiO_2_ nanosheets. This was achieved by treating TiO_2_ at 500 °C in a H_2_ atmosphere to create surface defects, such as oxygen vacancies (Vo), before depositing the Pt SA using a facile dark deposition/impregnation method. The optimized Pt-SA decoration resulted in a 150-fold increase in the normalized photocatalytic water splitting hydrogen evolution activity of a magnetron-sputtered TiO_2–*x*_ sample compared to a conventional platinum-nanoparticle-decorated TiO_2_ surface. Additionally, the anchoring of Pt SAs onto TiO_2_ nanosheets can be significantly improved by incorporating fluorine atoms into the lattice structure.^45^ Lee and colleagues developed a technique for synthesizing highly dispersed copper single atoms that exclusively occupy the most stable Ti vacancies in hollow TiO_2_ nanoparticles using a unique photoactivation strategy.^46^

TiO_2_ nanotubes are widely regarded as one of the most efficient nanostructures for photocatalysis due to several advantages. Their unique one-dimensional structure facilitates efficient electron transport, minimizing recombination losses and resulting in high photoconversion efficiency.^47,48^ Additionally, their large surface area provides numerous active sites for photocatalytic reactions, enhancing photon absorption and catalytic activity. Furthermore, nanotubes can be synthesized with well-controlled dimensions and surface properties, allowing for precise tuning of their photocatalytic properties.^3,35,49^ Regarding single-atom engineering on TiO_2_ nanotubes, it was demonstrated that their tube walls provide abundant surface Ti^3+^-Vo defects that are highly suitable trapping sites for highly effective extraction and accumulation of Pt in the form of single atoms (SAs).^50,51^

In this study, we propose a new technique for introducing lattice defects on pristine TNTs (titanium nanotubes), which act as a photocatalytically active support for tight and stable anchoring of highly active Pt-SA co-catalysts. The main innovation in this study represents the ability to precisely control surface reduction of TNTs through tunable and adjustable surface modification using ultrasonication treatment. Furthermore, introducing lattice defects in mild conditions by an ultrasound method can avoid the destructive effect of high temperature surface modification in a H_2_ atmosphere like sintering and phase transformation. Moreover, using the wet chemical method (impregnation) to form Pt-SA is straightforward and operable at any chemistry lab, which requires neither especial nor expensive instruments. Our findings showed that the well-dispersed and uniform Pt-SA decoration on the TNT support resulted in a 50-fold and 10-fold enhancement in the Pt-surface-amount-normalized photocatalytic activity for hydrogen evolution compared to those of the pristine and Pt-NP-decorated TNTs, respectively.

## 2 Experimental Details

### 2.1 Catalyst Synthesis

The electrochemical anodization experiments were performed in a standard two-electrode cell, with a titanium and a platinum foil as the working and the counter electrode, respectively. Prior to anodization, the Ti foils (0.25 mm, 99.5% purity, Sigma-Aldrich) were cut into pieces with 1.5 × 1.5 cm^2^ area, ultrasonicated with acetone, ethanol, and deionized water (DI) in sequence, and dried in nitrogen stream. After drying, the anodization was carried out at room temperature for 30 min by using a constant voltage of 70 V and an electrolyte solution containing 94.65 wt % ethylene glycol (99.5%, Sigma-Aldrich), 0.68 wt % HF (38–40%, Lachner), and 4.67 wt % deionized (DI) water (Sigma-Aldrich). The synthesized TNTs were then rinsed in DI water and subsequently calcined at 450 °C in air for 2 h with heating and cooling rate of 2 °C/min, and these samples are hereafter termed as pristine.

**Figure 1:**
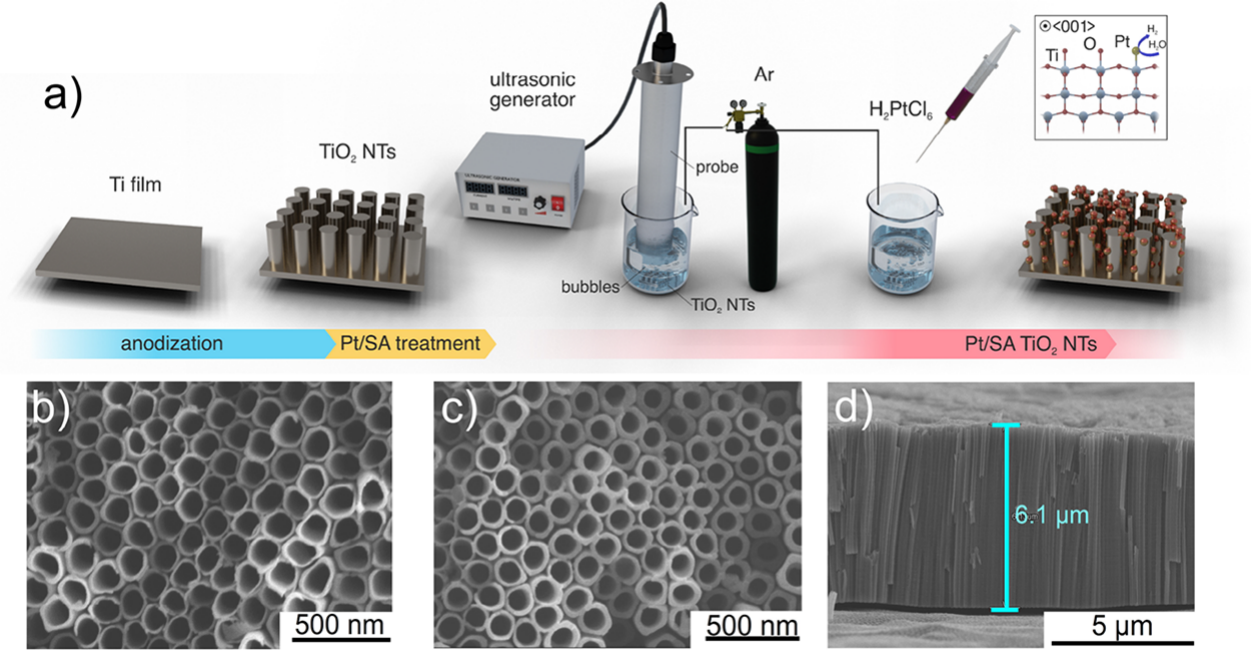
(a) Schematic illustration presenting the fabrication strategy of Pt-SA TNTs. (b) SEM image of pristine synthesized TNTs (P-NT). (c) SEM image of the sonicated sample for 50 min (R50-NT). (d) SEM cross section of P-NT.

Reduced TNTs were fabricated through sonicating the pristine TNTs for different sonication times (30, 40, 50, 60, 70, and 80 min) in a beaker filled with 100 mL of DI water and in an Ar atmosphere. The aqueous solution of the Pt SA deposition bath contained 100 μM chloroplatinic acid (H_2_PtCl_6_, Safina Ltd.) in a volume of 50 mL of DI water. Before soaking the TNT sample, the chloroplatinic acid solution was purged 30 min in Ar to remove the dissolved oxygen. Immediately after reduction using the sonication tip, to decorate Pt-SA, without any further purification, reduced TNTs were immersed in solution for different times (Figure 1a). For the sake of simplicity, pristine TNT arrays, sonicated TNTs, and Pt-decorated TNTs are hereafter referred to as P-NT, R*x*-NT (*x* = 30, 40, 50, 60, 70, and 80 min), and R50/Pt-NT*y* (*y* = 1, 10, 15, 30, and 60 min), respectively.

### 2.2 Characterization of Samples

The morphology of TNTs was visualized using a scanning electron microscope (SEM, Hitachi SU 6600). X-ray photoelectron spectroscopy (XPS) was performed using a PHI VersaProbe II (Physical Electronics) spectrometer using an Al Kα source (15 kV, 50 W) at room temperature (23 °C), under a partial vacuum (1.4 × 10^–7^ Pa). All of the achieved data were analyzed using a Multipack (Ulvac-PHI, Inc.) software package. High-resolution (HR) spectra of C 1s peaks were obtained by setting the pass energy to 23.500 eV and the step size to 0.200 eV where the binding energy values were corrected considering the C 1s peak (as a reference) at 284.8 eV. X-ray diffractometry studies (XRD, PANalytical) with a Co-Kα (λ = 1.54 Å) radiation source were carried out within the range of 20° ≤ 2θ ≤ 70° to investigate the crystalline structure of the synthesized TNTs. The microscopic features of the synthesized samples were investigated using a TEM JEOL 2010 with a LaB6-type emission gun, operating at 160 kV. High-resolution transmission electron microscopy (HRTEM) images and elemental maps were acquired with an FEI TITAN G2 60-300. The UV–Vis diffuse reflectance spectra of the fabricated TNTs were measured by means of an Analytic Jena (Model) spectrophotometer.

### 2.3 Photoelectrochemical Tests

The electrochemical impedance spectra (EIS) in a frequency range of 0.1 Hz to 100 KHz and photocurrent measurements were carried out with a standard three-electrode electrochemical cell using a Gamry potentiostat (series G300-Warmiste, PA, USA) in 1 M NaOH solution (pH = 13.6). The TNT samples were used as a working electrode with an active area of 0.28 cm^2^, Pt served as the counter electrode, and saturated Ag/AgCl (3 M KCl) was used as the reference electrode. A xenon lamp (*P* = 150 W) AM 1.5 G was used for illumination as the light source. The incident photon-to-current efficiency (IPCE) was measured using the xenon arc light source combined with a monochromator (Newport Oriel 1/8 Cornerstone). To evaluate the photoelectrochemical (PEC) water splitting activity of the fabricated samples, linear sweep voltammetry (LSV) measurement was performed under the simulated solar light illumination (1 sun, 1.5 AM G and 100 mW cm^–2^). Since the theoretical thermodynamic cell voltage to split the water is 1.23 V vs reversible hydrogen electrode (RHE) at 25 °C, the applied potential is converted from V vs Ag/AgCl to RHE scale using the following formula:
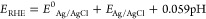
1where the pH is 13.6, *E*^0^_Ag/AgCl_ = 0.197 V at 298 K, and *E*_Ag/AgCl_ is the measured potential vs Ag/AgCl.

### 2.4 Photocatalytic H_2_ Evolution

The open-circuit photocatalytic H_2_ generation was assessed in a quartz reactor positioned in front of a solar simulator (1 sun, 1.5 AM G and 100 mW cm^–2^), and gas chromatography (GCMS-O2010SE, SHIMADZ) was used to detect the amount of generated hydrogen. Typically, the samples were immersed in 10 mL of aqueous solution containing 50 vol % methanol as a hole scavenger and DI water. Subsequently, the reactor was purged by Ar gas for 30 min to remove the oxygen, sealed with a rubber stopper, and then illuminated under 1 sun for 24 h.

The turnover frequency (TOF) is defined as
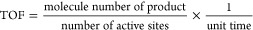
2

The TOF in single-atom catalysts can be expressed using the following equation:^51^
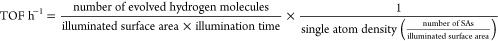
3

The illuminated surface area was 0.78 cm^2^.

## 3 Results and Discussion

The TNTs were fabricated by electrochemical anodization of titanium foil. As shown in Figure S1, the XRD pattern of the pristine nanotubes (P-NT) shows the formation of the anatase phase. Then, the samples were exposed to ultrasound post-treatment (140 W with a frequency of 18.1 KHz) for different sonication times (30, 40, 50, 60, 70, and 80 min) in an Ar atmosphere to form structural and electronic defects on the TNT surface (samples labeled as R30-NT to R80-NT). The ultrasonication treatment did not lead to any polymorphous transformations, and all the TNT sonicated samples maintained the anatase crystal structure (Figure S1). Figure 1b shows the SEM images of the annealed compact TNTs (P-NT) featuring a unique morphology with the open nanotubes at the top instead of common nanoporous layers terminating the nanotubes. We present the SEM image of R50-NT (the TNT sample sonicated for 50 min) as an exemplar morphology, which displays comparable open TNT morphology to P-NT (Figure 1c). As shown in Figure 1b,d, the mean diameter and the length of the P-NT sample were 100 nm and 6.1 μm, respectively.

**Figure 2:**
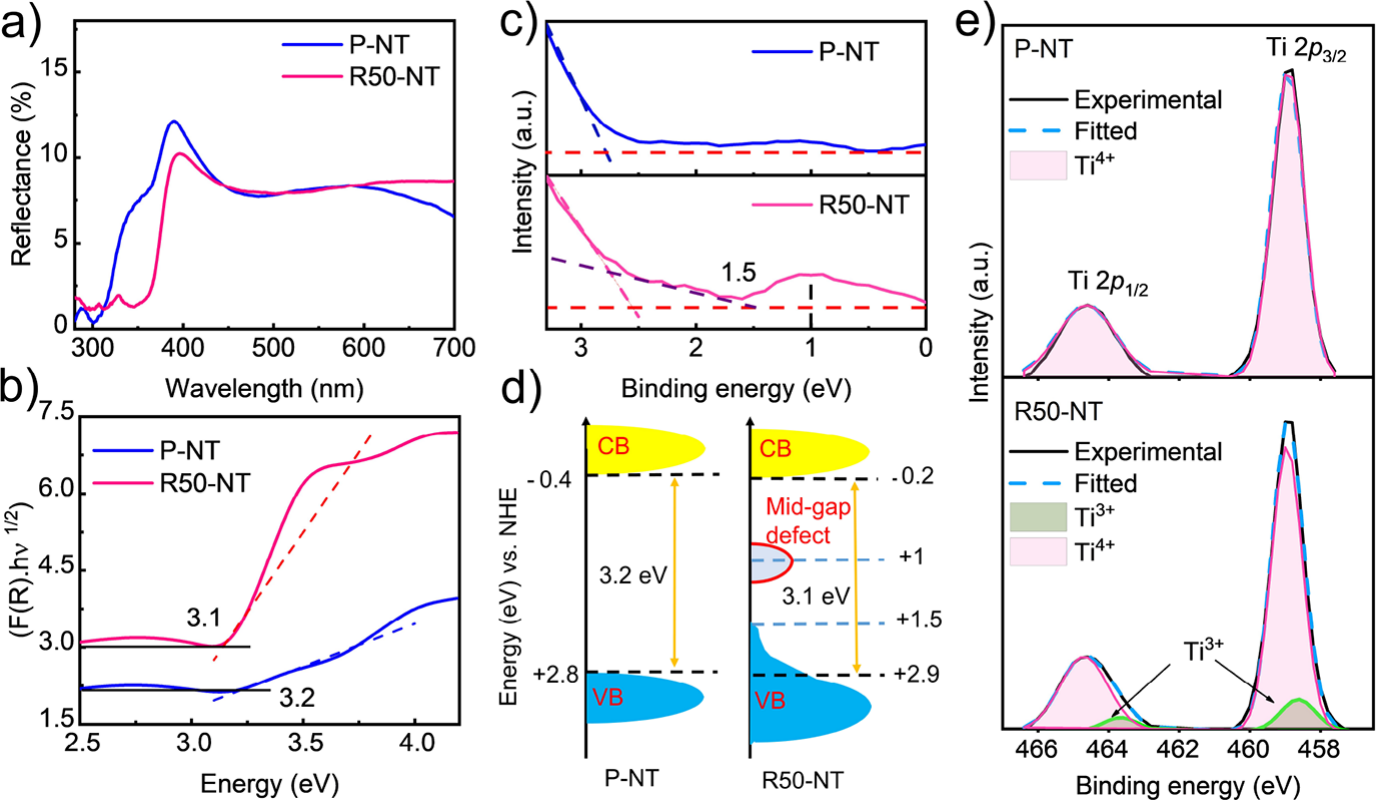
(a) UV–Vis DRS of P-NT and R50-NT, (b) calculated bandgap energies of P-NT and R50-NT, (c) XPS valence band spectra of P-NT and R50-NT, (d) schematic diagram of the DOS of P-NT and R50-NT, and (e) high-resolution XPS spectra in the Ti 2p region for P-NT and R50-NT.

To gain insights into the photophysical property enhancements of the sonicated sample (R50-NT), such as bandgap narrowing and the controlled creation of surface defects such as oxygen vacancies, we measured the occupied density of states (DOS) through the valence band (VB) XPS spectra and UV-DRS reflectance results (Figure 2a–d). By linear extrapolation of the peaks to the baseline, the valence band maxima can be retrieved. The P-NT sample showed the maximum energy of VB in DOS at +2.8 eV, and the maximum level of VB energy for the sonicated sample (R50-NT) was calculated at +2.9 eV followed by a band tail extending up to +1.5 eV (Figure 2d). Based on UV–Vis DRS measurements, the optical bandgap energies of the pristine TNTs (P-NTs sample) and sonicated one (R50-NT sample) are 3.2 and 3.1 eV, respectively. Consequently, the conduction band minima of R50-NT and P-NT samples were found to be located at −0.2 and −0.4 eV, respectively. Therefore, the bandgap narrowing observed for the sonicated sample can be attributed to the small tailing of the valence band. A combination of valence band XPS results with the optical bandgap measurement confirms that the sonication imprints the structural disorder and defects into the anatase crystal lattice, which is reflected in a shift of the valence band maxima and conduction band minima.^52^

Moreover, the results demonstrate that localized states can be formed above the valence band upon TNT reduction, as illustrated in Figure 2c,d. To further investigate the impact of sonication on the surface chemical composition and structural changes of the R50-NT sample (sonicated for 50 min), high-resolution XPS analysis was conducted (Figure 2e). The two broad peaks observed at 458.6 and 464.4 eV correspond to the Ti2p_3/2_ and Ti2p_1/2_ orbitals of Ti^4+^ in TNT lattice, respectively.^53,54^ The two less intense peaks at lower binding energies observed in the R50-NT sample, which contains defects, can be attributed to Ti^3+^ species.^55,56^ In contrast, the pristine NT sample only displayed peaks related to the Ti2p_3/2_ and Ti2p_1/2_ orbitals of Ti^4+^.

**Figure 3:**
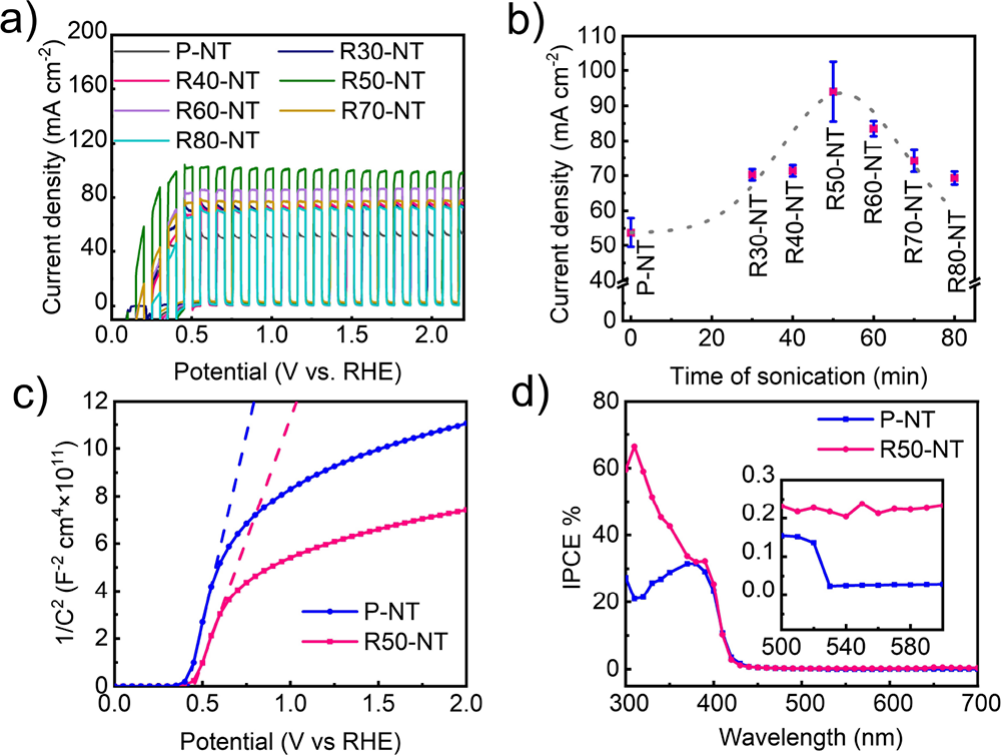
(a) Photoelectrochemical response of TNT samples sonicated for different times measured under 1 sun illumination (100 mW cm^–2^, AM1.5 G) in 1 M NaOH solution. (b) Comparison of current densities at 1.23 V for the investigated samples. (c) Mott–Schottky plots of P-NT and R50-NT obtained at a frequency of 5 KHz in the dark and (d) corresponding IPCE spectra.

To identify the most efficient sample for Pt SA-trapping, the photoelectrochemical (PEC) water splitting activity of sonicated samples with varying sonication times was estimated. The measurements of current density versus applied voltage (*J*–*V*), i.e., linear voltammetry experiments, were carried out in the dark and light conditions using AM 1.5 G illumination (100 mW cm^–2^). The PEC investigation results revealed that all sonicated samples exhibit higher PEC activity than the pristine one (P-NT) due to the defect formation, which improves donor density and charge carrier transport (Figure 3a,b).^57,58^ Nevertheless, the relationship between current density and sonication time is non-monotonic, with the maximum observed in the R50-NT sample (Figure 3b). This suggests that there is an optimal degree of defect and sonication time for achieving maximum PEC efficiency and that longer sonication times do not lead to further enhancements. Based on the observed results, the sample sonicated for 50 min (R50-NT) displayed the highest anodic photocurrent density of 100 μA cm^–2^ at 1.23 V under solar irradiation, indicating the highest PEC activity. As a result, this sample was selected for subsequent decoration with platinum single atoms.

The Mott–Schottky plots of the P-NT and R50-NT electrodes (Figure 3c) exhibited positive slopes for both samples, indicating an n-type semiconductor behavior.^59^ In addition, the carrier density was determined using the following equation:
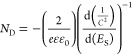
4where *C* is the space charge capacitance for the semiconductor, *N*_D_ is the carrier density of electrons, *e* is the electrical charge, ε_0_ is the permittivity of the vacuum, ε is the relative permittivity of the semiconductor (TNTs), *K*_B_ is the Boltzmann constant, *E*_S_ is the applied potential, *E*_FB_ is the flat potential, and *T* is the absolute temperature. Here, *e* = −1.6 × 10^–19^, ε_0_ = 8.86 × 10^–12^ Fm^–1^, and ε = 48 for TNTs in the anatase phase.^60^ The carrier density values obtained for R50-NT and P-NT were 1.43 × 10^16^ cm^–3^ and 0.92 × 10^16^ cm^–3^, respectively. This indicates that the sonication reduction process led to an increase in electron density in the R50-NT sample (sonicated sample for 50 min) by introducing defects such as oxygen vacancies and Ti^3+^ states.^61^ The electrochemical impedance measurement was conducted under 1 sun illumination at a bias voltage +1.5 vs RHE in the range of 0.1 Hz to 100 KHz (100 mW cm^–2^). The semicircle radius obtained from the Nyquist diagram of EIS data (Figure S2) for the R50-NT sample is smaller than P-NT, indicating lower resistance to charge transfer at the electrode/electrolyte interface.^3^ As a result, sonicated TNTs (R50-NT) exhibited higher PEC activity due to the higher concentration of electron donors (*N*_D_) and promotion of faster charge transfer (lower resistivity). The incident photon to current efficiency (IPCE) spectra for the two selected tested samples (i.e., P-NTs and R50-NT) were measured at +1.5 V vs RHE in a 1 M NaOH solution to determine the contribution of each wavelength to the photocurrent density. The IPCE can be calculated using the following formula:^49^
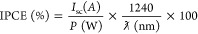
5where ƛ is the incident light wavelength (nm), *I* is the measured photocurrent density (μA cm^–2^), and *P* is the measured irradiation of incident light at a specific wavelength. The R50-NT sample exhibited significantly higher IPCE values in the UV region, reaching approximately 70% at 380 nm, compared to the 50% for the P-NT sample (Figure 3d). This indicates that efficient separation of photoexcited charge carriers is mainly improved for UV-promoted electronic transitions, i.e., those associated with VB to CB transitions.^54^ Notably, a relatively small increase in the photocurrent density of R50-NT can be observed throughout the visible region (inset Figure 3d), which can be attributed to the higher light absorption in this region (Figure S3 and Figure 2a).^62,63^

**Figure 4:**
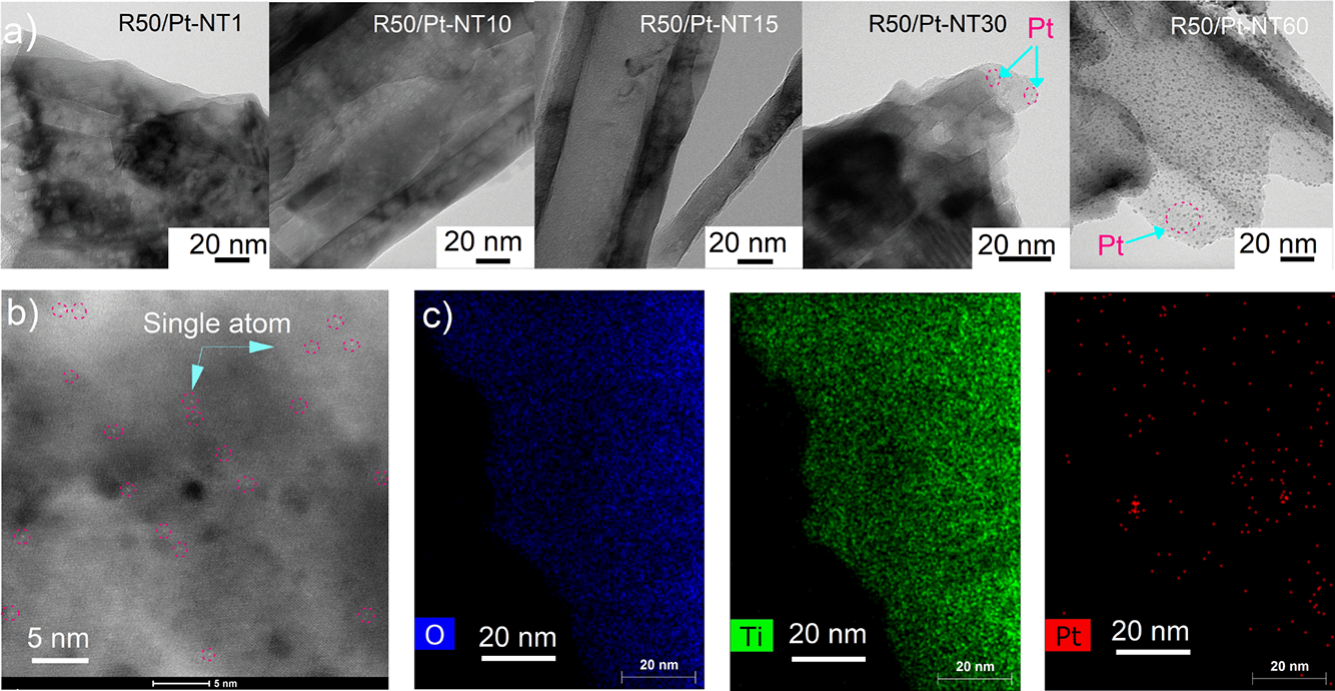
(a) TEM images of treated samples at different impregnation times (ranging from 1 to 60 min) in hexachloroplatinic acid solution. (b) HAADF-STEM image of the R50/Pt-NT10 sample. (c) EDS elemental mapping of the R50/Pt-NT10 sample.

The Pt SAs were deposited on the optimally sonicated R50-NT sample by its impregnation in a 100 μM hexachloroplatinic acid solution. To investigate the effectiveness of the impregnation process at different durations, five samples were prepared, each impregnated for a different amount of time: 1, 10, 15, 30, and 60 min. These samples were labeled as R50/Pt-NT1, R50/Pt-NT10, R50/Pt-NT15, R50/Pt-NT30, and R50/Pt-NT60, respectively. Figure 4a presents the TEM micrographs of all Pt-loaded samples. The samples impregnated for 1–15 min do not indicate the presence of Pt nanoparticles, while samples impregnated for longer times (R50/Pt-NT30 and R50/Pt-NT60) contain ultrasmall nanoparticles (Figure 4a). This is in line with the results of energy-dispersive spectrometry (EDS) analysis (Figure S4a), which does not detect Pt in samples with impregnation times less than 15 min (due to a very low content of Pt). Furthermore, Figure S4b displays a very narrow particle size distribution (1–2 nm) and an average diameter about 1.5 nm for Pt nanoparticles formed in the sample impregnated for the longest time (R50/Pt-NT60). To corroborate the presence of Pt-SA on the surface of TNTs impregnated for a short impregnation time, high-angle-annular-dark-field scanning transmission electron microscopy (HAADF-STEM) of R50/Pt-NT10 sample was performed (Figure 4b). The large number of Pt single atoms (marked bright dots) was clearly identified on the surface of the R50/Pt-NT10 sample. To assess the distribution of Ti, O, and Pt over the catalyst, EDS mapping was carried out. Figure 4c and Figure S5 present the EDS map of Pt, Ti, and O elements of the R50/Pt-NT10 sample, which further confirms the almost uniform distribution of elements, including the presence of Pt single atoms; no contamination was observed.

**Figure 5:**
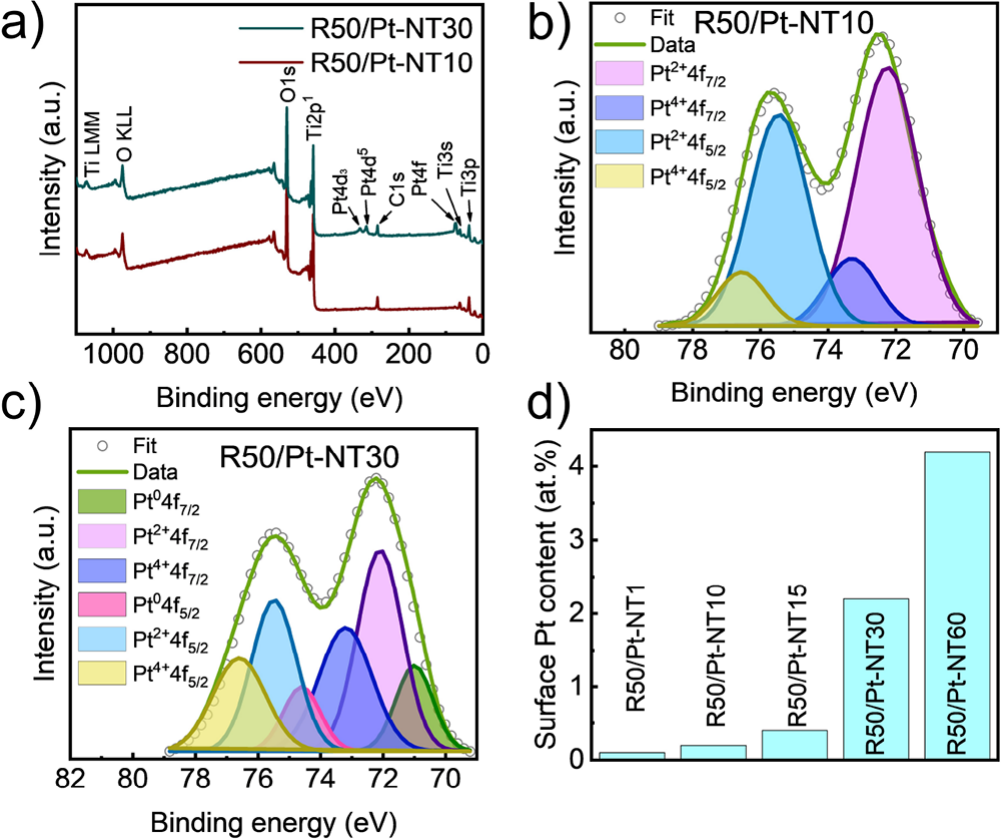
(a) Representative XPS survey spectra of R50/Pt-NT30 and R50/Pt-NT10 samples. (b) HR XPS spectra of Pt 4f for the R50/Pt-NT10 sample containing just Pt single atomic sites. (c) HR XPS spectra of Pt 4f for the R50/Pt-NT30 sample containing both Pt single atomic sites and Pt nanoparticles. (d) Surface Pt content for Pt-impregnated samples as determined by XPS.

To characterize the structural and chemical composition of the Pt-loaded samples, the XPS analysis was performed. The spectra of Pt-loaded samples impregnated for different soaking times are illustrated in Figure 5a–c and Figure S6a–d. From the survey spectrum of R50/Pt-NT30, the elements Ti, O, Pt, and adventitious C can be observed (Figure 5a) and the atomic ratio of Ti:O corresponds to the expected composition of TiO_2_ (1:2). The deconvoluted high-resolution XPS (HR XPS) spectra of the Pt 4f region for the single atoms (R50/Pt-NT10) and Pt nanoparticle-decorated (R50/Pt-NT30) samples are displayed in Figure 5b,c, respectively. Figure 5b demonstrates that the XPS spectrum of Pt 4f for the R50/Pt-NT10 sample (Pt-SA sample soaked for 10 min in hexachloroplatinic acid solution) can be fitted into four peaks. The peaks that appear at 72.3 and 75.7 eV can be attributed to Pt^2+^4f_7/2_ and Pt^2+^4f_5/2_, respectively, whereas the peaks at 73.2 and 76.5 eV are assigned to Pt^4+^4f_7/2_ and Pt^4+^4f_5/2_ signals. These data reveal that the R50/Pt-NT10 sample contain both Pt^4+^ and Pt^2+^ single atomic sites, while zero-valent metallic species are not detected. By contrast, it can be observed from the XPS spectrum of the sample obtained by 30 min impregnation time in hexachloroplatinic acid solution (R50/Pt-NT30) in Figure 5c that the region corresponding to the Pt peak may be deconvoluted into three components (Pt^2+^, Pt^4+^, and Pt^0^). The two peaks located at 71.1 and 74.5 eV are corresponding to the metallic state Pt^0^4f_7/2_ and Pt^0^4f_5/2_, respectively.^23,39,64^ These results are in good agreement with the TEM results (Figure 4a) where nanoparticle formation can be observed only after 30 min impregnation time in hexachloroplatinic acid solution. The presence of Pt^4+^ coming from the covalent metals–support interaction is a proof of a strong Pt-TiO_2_ interaction.^23,65^ The Pt-4O and Pt-2O structures are the most stable geometric and electronic configurations of platinum bonded to oxygen, which are also stable against the initial states of agglomeration.^66^ Therefore, they confirm the atomic-scale deposition of single atom Pt on the sonicated TNT surface (R50/Pt-NT10 sample). The HR XPS analysis of R50/Pt-NT1 and R50/Pt-NT15 proved the presence of Pt exclusively in the ionic state (Pt^2+^ and P^+4^), as can be seen in Figure S6b,c. Moreover, the HR XPS spectra of Pt4f for these two samples (R50/Pt-NT1 and R50/Pt-NT15) are observed to be quite similar to that of the Pt4f peaks of the R50/Pt-NT10 sample, meaning that all of these three samples are in the Pt-SA nature. In contrast, for the samples R50/Pt-NT60 (Figure S6d), the Pt species in the metallic state (Pt^0^) with peaks located at 70.2 and 73.5 eV are observed and can be assigned to Pt^0^4f_7/2_ and Pt^0^4f_5/2_, respectively.^67,68^ The XPS results of the decorated samples with Pt are in line with the TEM images, confirming that the concentration of stabilized surface Pt is adjustable by increasing the soaking time in a diluted hexachloroplatinic acid solution. As the ICP-mass measurement for the film sample was very challenging to quantify the exact amount of the Pt species on the TNT film, XPS measurement was applied.^23^ As a result, the concentrations of Pt in atomic-scale form for the R50/Pt-NT1, R50/Pt-NT10, and R50/Pt-NT15 samples are approximately 0.1, 0.2, and 0.4 at %, respectively. Meanwhile, the amounts of Pt in the form of both single atoms and nanoparticles, based on XPS results, are 2.2 and 4.2 at % for the R50/Pt-NT30 and R50/Pt-NT60 samples, respectively (Figure 5d). Interestingly, as shown in Figure S7**,** the peak area corresponding to Ti^3+^ gradually vanished in the HR XPS spectra after impregnation with hexachloroplatinic acid solution. This can be the result of incorporation of Pt single ionic species into the oxygen vacancies and charge re-distribution in the local environment of Pt single atoms.

**Figure 6:**
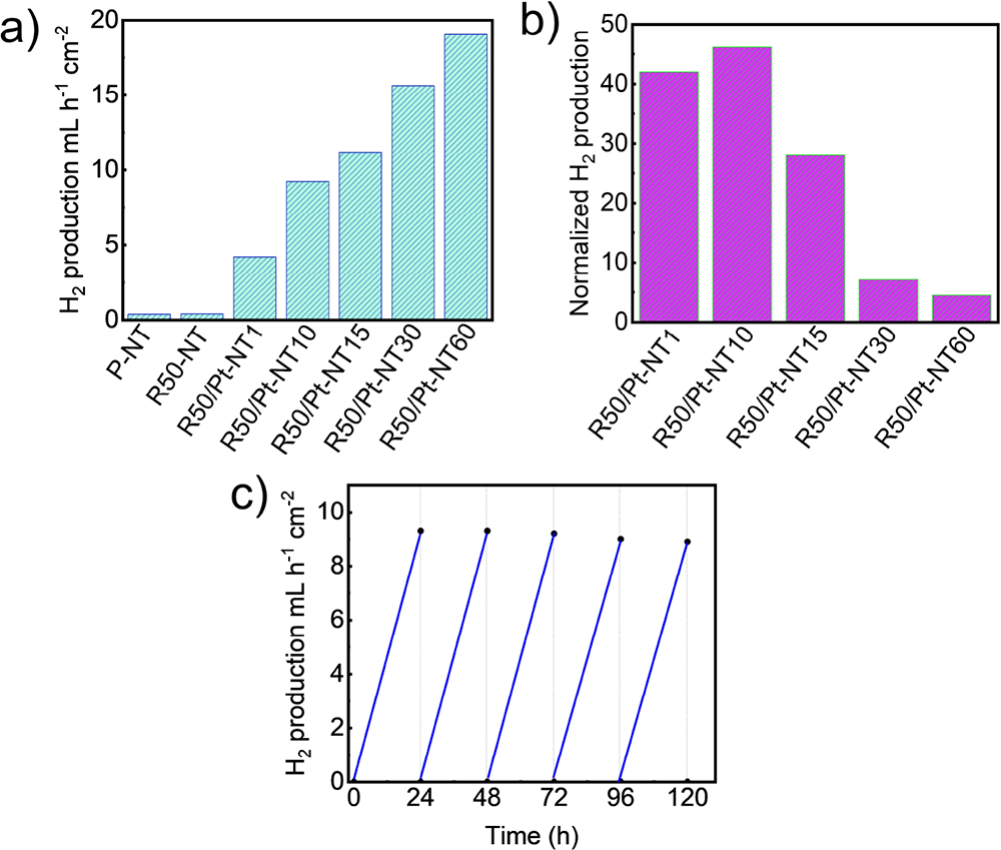
(a) Hydrogen evolution of pristine, sonicated, and Pt-decorated TNTs. (b) Normalized H_2_ evolution of SA-Pt catalysts. (c) Reusability test of H_2_ evolution of R50/Pt-NT10—the sample sonicated for 50 min followed by immersion in hexachloroplatinic acid solution for 10 min.

To evaluate the photocatalytic hydrogen production of ultrasound-pretreated and platinized TNTs, the H_2_ evolution (Figure 6a) was investigated in methanol–water solution using 1 sun solar simulation (AM 1.5 G-100 mW cm^–2^). Under light illumination, all the Pt-decorated TNTs revealed a higher H_2_ evolution rate compared to the pristine and sonicated TNTs without Pt (nearly 0.4 μL h^–1^ cm^–2^ for P-NT and R50-NT samples). In fact, the hydrogen evolution of Pt-decorated samples (for the lowest amount of Pt in R50/Pt-NT1) is at least 10 times higher than the value obtained for pristine TNTs sample (P-NT). Likewise, by increasing the impregnation time of sonicated samples in the dilute hexachloroplatinic acid solution, the hydrogen evolution rate increased because of the higher loading of Pt on the surface of sonicated TNTs. This indicates that both single atom platinum sites and Pt nanoparticles contribute to the increased hydrogen evolution. However, normalization to the surface amount of Pt (Figure 6b) shows that the normalized hydrogen evolution is significantly higher for samples with the exclusive presence of single atom Pt sites, i.e., for samples impregnated for short times (R50/Pt-NT1 and R50/Pt-NT10). Here, the hydrogen evolution is ca. 40–50 times higher compared to the non-impregnated samples. The gradual decrease in normalized hydrogen evolution with impregnation time (Figure 6b) reflects the increasing content of platinum nanoparticles at the expense of single-atom platinum sites. This enables a direct comparison of the efficiency of single atomic platinum sites vs ultrasmall platinum nanoparticles. This comparison is very objective as both single-atom sites and nanoparticles are formed in the same chemical system by the same chemical strategy. From this comparison, it can be extracted that the efficiency of platinum single atoms is ca. one order of magnitude higher (sample R50/Pt-NT10) compared to Pt nanoparticles (R50/Pt-NT60).

The efficiency of the single-atom active catalytic centers can be expressed as the turnover frequency (TOF) number. The TOF is calculated using Pt single atom density from the HAADF-STEM image (Figure S8) and the evolved H_2_, resulting in a record value of 2.9 × 10^7^ h^–1^. This number obtained by ultrasound-driven defect engineering is one order of magnitude higher than that obtained on nanotubes with defect induced by thermal treatment.^51^ The stability and reusability of the single-atom platinum-containing samples were tested with a R50/Pt-NT10 sample (Figure 6c). No obvious decrease in H_2_ production after five successive cycles was observed, which indicates the strong bonding of single atoms and a good stability of synthesized photocatalysts.

**Figure 7:**
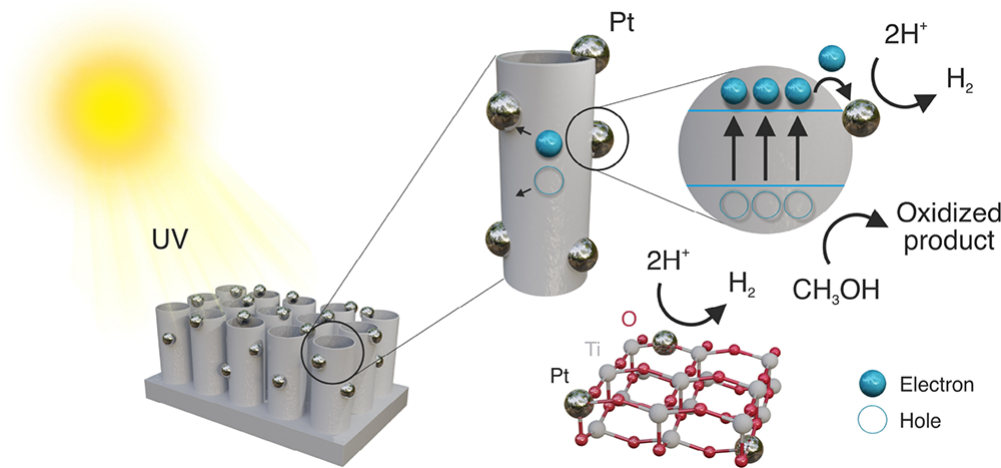
Mechanism of photocatalytic hydrogen evolution using ultrasound-reduced TNTs with embedded Pt single-atom co-catalysts.

Figure 7 demonstrates the schematic illustration of the reaction of photocatalytic H_2_ production through illumination of Pt SA-TiO_2_. When the energy of the irradiated light is larger than the bandgap of TiO_2_, photons are absorbed by TiO_2_ to form electron–hole pairs. Photogenerated electrons–holes can be separated into the conduction (CB) and valence (VB) bands, respectively. The methanol solution acts as a sacrificial reducing agent reacting with photogenerated holes, which hinder the charge carrier recombination rate. Finally, Pt plays a key role as the reductive co-catalyst reducing H^+^ to H_2_, as shown in Figure 7.^69^

## 4 Conclusions

In conclusion, we have shown for the first time, single atomic-scale Pt sites on the defective TNT array film using the state-of-the-art technique to form lattice defects on the surface of the substrate. Ultrasonication was employed to form oxygen vacancy/Ti^3+^ states on the surface of TNTs, which were confirmed by HR XPS, UV–vis DRS, and Mott–Schottky measurements. Such defects result not only in the shift of the maxima of the valence band of TiO_2_ upward for bandgap narrowing but also some mid-gap defect might be formed. The results demonstrated that the time of impregnation plays a major role in controlling Pt-SA decoration on the surface of TNTs. According to this finding, the optimized soaking time produced single Pt atoms confirmed by the presence of Pt^2+^ and Pt^4+^ species that enabled a photocatalytic H_2_ evolution rate nearly 10-fold higher than that of the TNTs decorated with Pt NPs. The developed approach opens the way to an effective immobilization of single atomic species on metal oxide surfaces without applying any thermal reduction treatment, thus producing very active photocatalysts for hydrogen evolution. The ultrasound-driven precise defect engineering brings high controllability over the process and avoids the harsh reaction conditions in common reduction methods at high temperatures such as phase transformation, sintering, impurities, and even peeling-off the fabricated nanotubes. On the other side, scaling up this technique to very large samples still remains challenging.

## Supporting Information Available

XRD patterns of pristine and reduced TNTs, EIS spectra, plot of Kubelka–Munk reflectance, EDS of Pt-decorated TNTs and Pt nanoparticle distribution, EDS elemental mapping of the Pt-SA sample, XPS survey spectra of Pt-SA and Pt-NP samples, HR XPS spectra in the Ti 2p region of Pt-decorated TNTs, and HAADF-STEM image for SA calculation area (PDF)
