# Supplementary material for: Ultrasound-Driven Defect Engineering in TiO2–x Nanotubes—Toward Highly Efficient Platinum Single Atom-Enhanced Photocatalytic Water Splitting
Source: ACS Appl Mater Interfaces. 2023 Jul 25;15(31):37976–85. doi: 10.1021/acsami.3c04811 (PMC10416212; doi:10.1021/acsami.3c04811)
Supplement: Supplementary file 1 — am3c04811_si_001.pdf [file am3c04811_si_001.pdf]

Supporting Information

Ultrasound-driven defect engineering in TiO_{2-x} nanotubes – towards highly efficient platinum single atom enhanced photocatalytic water splitting

Mahdi Shahrezaei[a,b], S. M. Hossein Hejazi[a,c], Hana Kmentova[a], Veronika Sedajova[a], Radek Zboril[a,c], Alberto Naldoni[d*], and Stepan Kment[a,c*]

[a] Czech Advanced Technology and Research Institute, Regional Centre of Advanced Technologies and Materials, Palacký University Olomouc, Slechtitelu 27, 77900 Olomouc, Czech Republic

[b] Department of Physical Chemistry, Faculty of Science, Palacky University, 17. listopadu 1192/12, 779 00 Olomouc, Czech Republic

[c] CEET, Nanotechnology Centre, VŠB–Technical University of Ostrava, 17. listopadu 2172/15, 708 00 Ostrava-Poruba, Czech Republic

[d] Department of Chemistry and NIS Centre, University of Turin, Turin 10125, Italy

*Corresponding author: stepan.kment@upol.cz; alberto.naldoni@unito.it

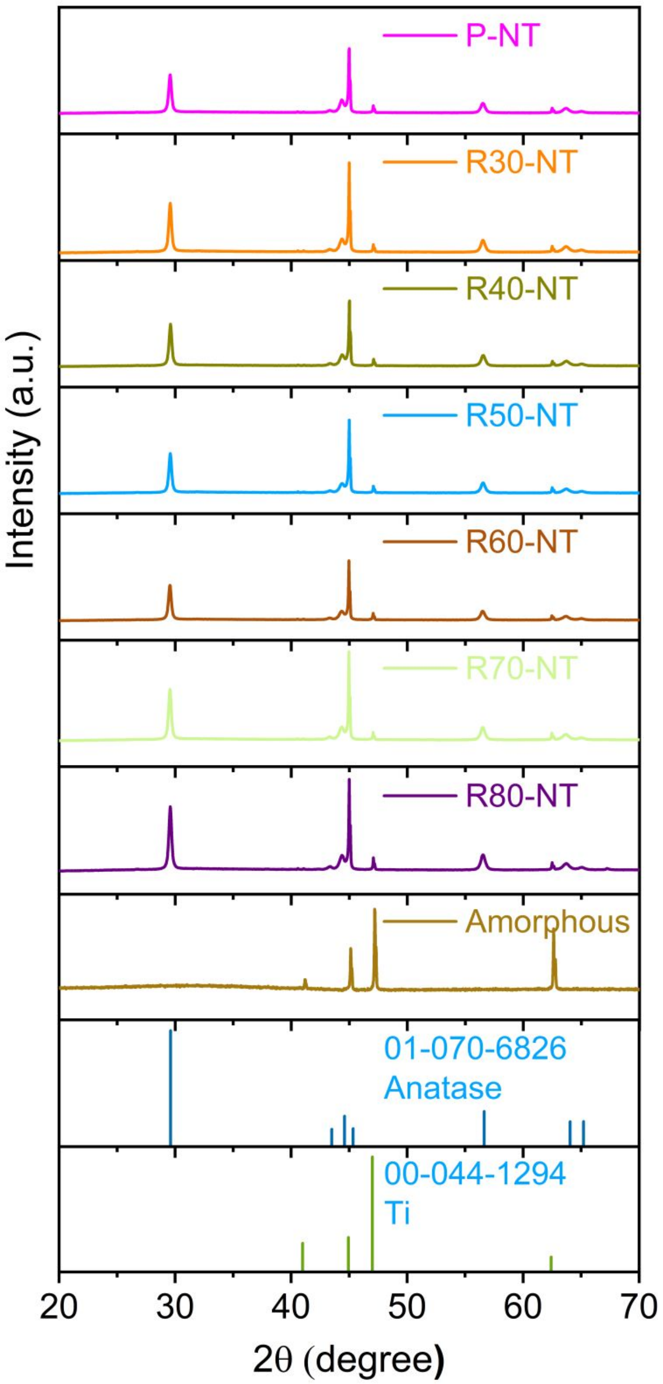

Figure S1. XRD patterns of pristine and reduced TNTs using a cobalt X-ray source (P-NT is pristine TNTs and R30-NT is sonicated TNTs for 30 min etc.

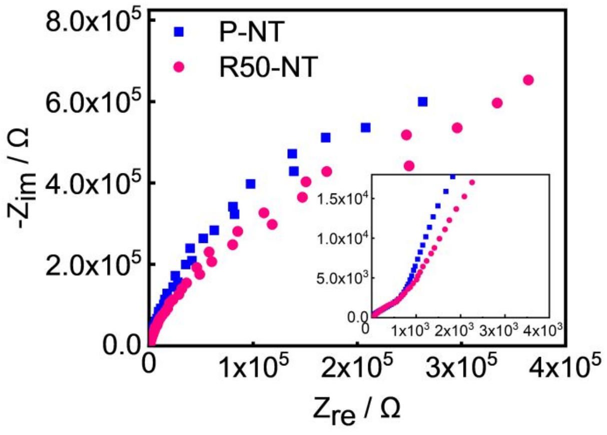

Figure S2. Nyquist plots representing electrochemical impedance spectra of P-NT and R50-NT samples.

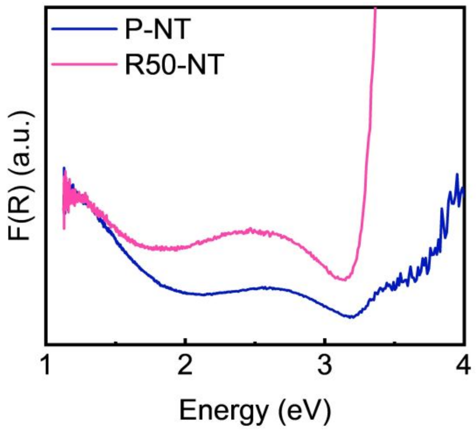

Figure S3. Plot of Kubelka-Munk reflectance vs. energy for pristine and sonicated sample for 50 min (P-NT and R50-NT respectively).

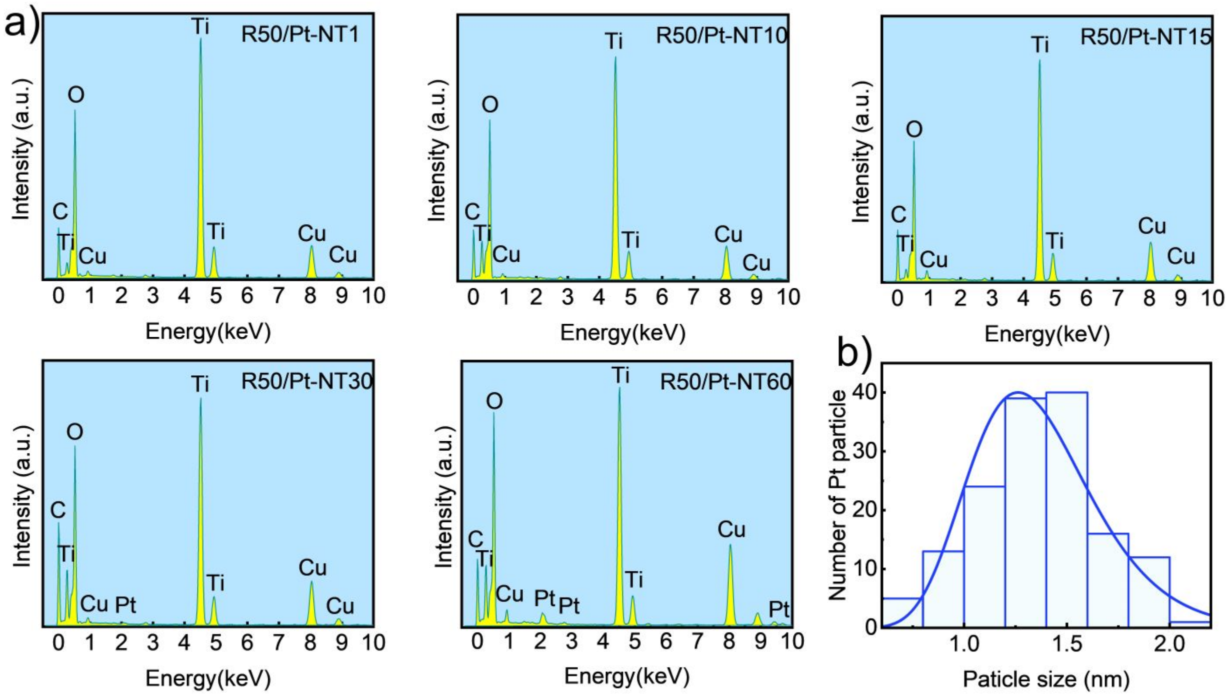

Figure S4. a) EDS of Pt-SA and Pt-NP decorated TNTs and b) Nanoparticles distribution of Pt on TNTs for R50/Pt-NT60 sample.

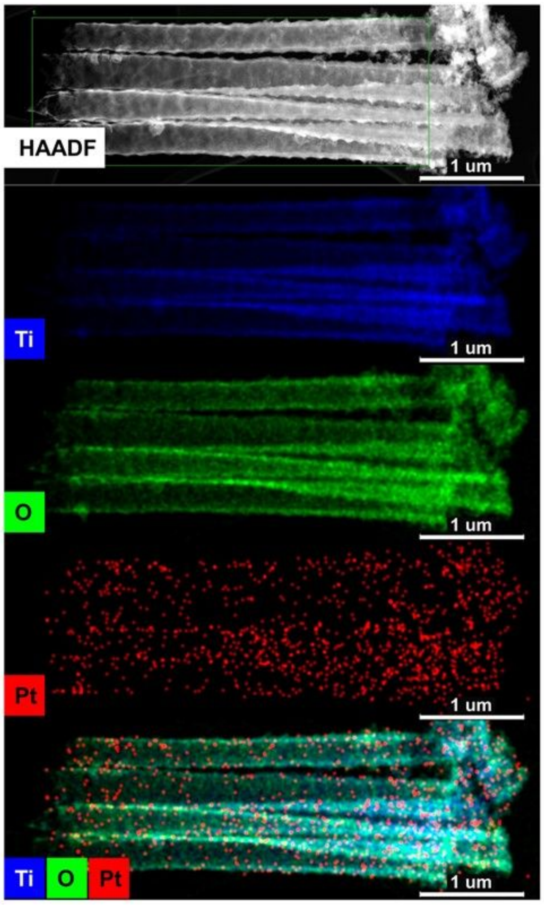

Figure S5. EDS elemental mapping of R50/Pt-NT10 sample.

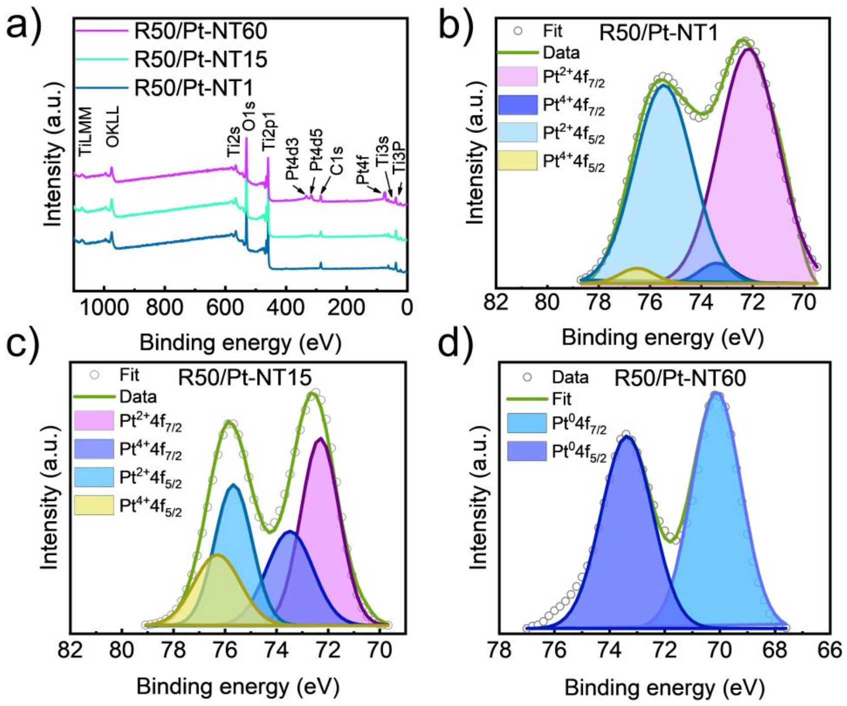

Figure S6. a) Representative XPS survey spectra of R50/Pt-NT1, R50/Pt-NT15 and R50/Pt-NT60 samples, b, c) HR XPS spectra of Pt4f for R50/Pt-NT1 and R50/Pt-NT15 samples, respectively, containing just Pt single atomic, and d) HR XPS spectra of Pt4f for R50/Pt-NT60 containing Pt nanoparticles.

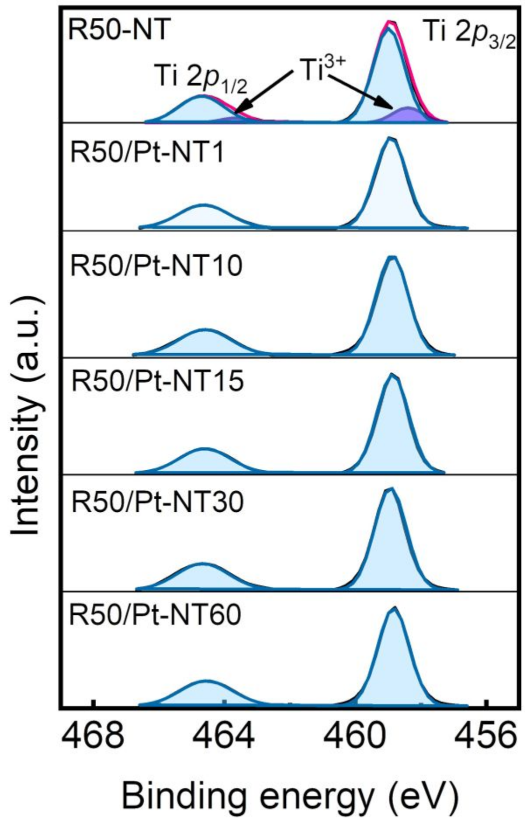

Figure S7. HR XPS spectra in the Ti2p region of sonicated TNTs for 50 min, followed by immersion in the Pt solution for 1, 10, 15, 30 and 60 min (R50/Pt-NT1, R50/Pt-NT10, R50/Pt-NT15, R50/Pt-NT30 and R50/Pt-NT60).

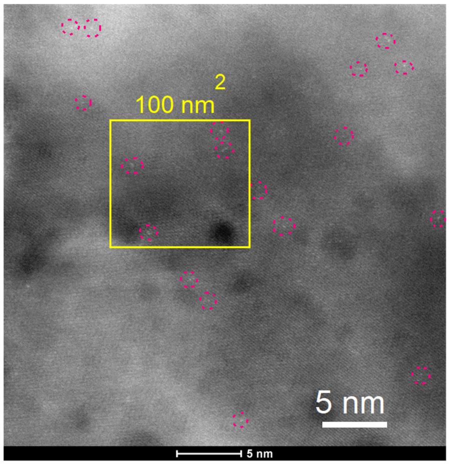

Figure S8. HAADF-STEM image of R50/Pt-NT10 and corresponding SA calculation area (10 nm×10 nm).
